# Editorial Comment: How I do it open distal ureteroureterostomy for ectopic ureters in infants with duplex systems and no vesicoureteral reflux under 6 months of age

**DOI:** 10.1590/S1677-5538.IBJU.2020.0742.1

**Published:** 2021-03-25

**Authors:** Ubirajara O. Barroso

**Affiliations:** 1 Universidade Federal da Bahia Escola Bahiana de Medicina Disciplina de Urologia SalvadorBA Brasil Disciplina de Urologia, Universidade Federal da Bahia e Escola Bahiana de Medicina Salvador, BA, Brasil.

## COMMENT

Ipsilateral ureterourethrostomy (IUU) is not a new procedure. It was first described by Foley in 1928, with laterolateral ureteral anastomosis and by Butchel in 1965 with terminolateral anastomosis (1, 2). In 1979, Bracci et al published a study on 25 patients that included cases of ureterocele, vesicoureteral reflux to the lower unit, and ectopic ureter, which were treated with IUU without stenting (3). Since then, this procedure has gained popularity and has become the procedure of choice in cases of functioning upper pole kidneys (UPK).

It has been demonstrated that non-functioning UPK are unable to recover function by histological criteria (4). Therefore, for non-functioning UPK the standard approach has become upper pole nephrectomy. Further justifications for upper pole nephrectomy has been the supposed increased risk of hypertension and tumors when the dysplastic kidney is left in situ. Two articles, however, have challenged this concept. Levy et al found no association between hypertension and the preservation of the UPK (5). After 15 years of follow-up, 9% and 8% of patients treated with upper pole nephrectomy and reconstruction of the lower urinary tract, respectively, developed arterial hypertension. In another study, Gran et al. performed ureteral reimplantation in 16 patients with duplex kidneys and ureterocele and no function of the UPK (6). They did not observe hypertension or tumors in the mean follow-up of 62 months and therefore proposed that a lower tract reconstruction by means of ureteral reimplantation could be the procedure of choice.

Because IUU is much less invasive than the ureteral reimplantation, many surgeons including myself begun to perform this procedure for patients with non-functioning UPK. Chacko et al. in 2007 were the first to publish the use of IUU for any type of obstructive duplex system, including those with functioning and non-functioning UPK (7). Prieto et al, in 2009, confirmed the encouraging results with this technique using a hidden hernia incision (8). Some pediatric urologists maintain that a disadvantage of upper polar nephrectomy is the possibility of some loss of function of the lower unit in 17% of cases (9). However, simplified renal nephrectomy practically avoids this problem (10) and, therefore, I do not believe that this is a significant argument. Nevertheless, in my view, there are other advantages of IUU over upper pole nephrectomy. IUU is an extra-peritoneal surgery, which can be performed as an outpatient procedure, with a hernia incision of 2 to 2.5 cm, minimal pain, without access to abdominal organs or large vessels, and with no risk of bleeding. In addition, since IUU is performed distally close to the bladder, the excess of the ureteral stump is easily removed, even if it infrequently causes complications (11).

Possible disadvantages of IUU include Yo-Yo reflux, anastomosis stricture, and the need for double J, which is known to cause urinary tract infection. The importance of Yo-Yo reflux has not been clearly demonstrated in studies; anastomosis stricture is quite uncommon; and some have performed the procedure without double J. I still prefer to use double J in the lower unit and use strings to remove it in the office in a week. IUU can also be performed by laparoscopy or robotics, I do not see any advantage for doing so with younger children.

Wang and Braga (12), in a didactic and elegant way, have illustrated the main steps of the IUU technique. As shown in the pictures, it is possible to perform the procedure even regardless of the diameter of the ureter. The photo shows a case with a Gibson incision, but even smaller and lower horizontalized incisions in the inguinal area can be performed. Wang and Braga’s article helps to popularize this technique. Although IUU is gaining more and more popularity and is quite attractive, it is important to emphasize that there are no randomized studies that compare this technique with upper pole nephrectomy.
